# Reliability and Validity of UNESP-Botucatu Cattle Pain Scale and Cow Pain Scale in *Bos taurus* and *Bos indicus* Bulls to Assess Postoperative Pain of Surgical Orchiectomy

**DOI:** 10.3390/ani13030364

**Published:** 2023-01-20

**Authors:** Rubia M. Tomacheuski, Alice R. Oliveira, Pedro H. E. Trindade, Flávia A. Oliveira, César P. Candido, Francisco J. Teixeira Neto, Paulo V. Steagall, Stelio P. L. Luna

**Affiliations:** 1 Department of Surgical Specialties and Anesthesiology, Botucatu Medical School, São Paulo State University (UNESP), Botucatu 18618-687, SP, Brazil; 2 Department Veterinary Surgery and Animal Reproduction, School of Veterinary Medicine and Animal Science, São Paulo State University (UNESP), Botucatu 18618-681, SP, Brazil; 3 University Veterinary Clinic, School of Veterinary Medicine and Animal Science, Federal University of Northern Tocantins, Araguaína 77804-970, TO, Brazil; 4 Department of Veterinary Clinical Sciences and Centre for Companion Animal Health and Welfare, Jockey Club College of Veterinary Medicine and Life Sciences, City University of Hong Kong, Hong Kong SAR, China; 5 Department of Clinical Sciences, Faculty of Veterinary Medicine, Université de Montréal, Saint-Hyacinthe, QC J2S 2M2, Canada

**Keywords:** animal behaviour, animal welfare, domestic cows, farm animals, pain assessment, pain measurement

## Abstract

**Simple Summary:**

This study aimed to investigate the reliability and validity of the UNESP-Botucatu cattle pain scale (UCAPS) and the cow pain scale (CPS) for postoperative pain assessment in *Bos taurus* (Angus) and *Bos indicus* (Nelore) bulls undergoing general anaesthesia and castration. Video recording performed for 3 min at five different time points (M0 and M1 preoperative; M2, M3 and M4 postoperative), resulting in 95 randomised videos, were assessed by two evaluators in two phases. The pain was assessed with UCAPS, CPS, a numerical rating scale (NRS) and a visual analogue scale (VAS). There were no significant differences in the scores of any scale between breeds. Intra- and inter-rater reliability varied from good (>0.70) to very good (>0.81). The UCAPS and CPS were responsive, specific (81–85%) and sensitive (82–87%). The cut-off point for rescue analgesia was >4 for UCAPS and >3 for CPS. Both instruments are valid and reliable instruments to assess postoperative pain in *Bos taurus* and *Bos indicus* bulls. The defined cut-off point of both scales can guide decision-making for rescue analgesia.

**Abstract:**

Pain assessment guides decision-making in pain management and improves animal welfare. We aimed to investigate the reliability and validity of the UNESP-Botucatu cattle pain scale (UCAPS) and the cow pain scale (CPS) for postoperative pain assessment in *Bos taurus* (Angus) and *Bos indicus* (Nelore) bulls after castration. Methods: Ten Nelore and nine Angus bulls were anaesthetised with xylazine–ketamine–diazepam–isoflurane–flunixin meglumine. Three-minute videos were recorded at -48 h, preoperative, after surgery, after rescue analgesia and at 24 h. Two evaluators assessed 95 randomised videos twice one month apart. Results: There were no significant differences in the pain scores between breeds. Intra and inter-rater reliability varied from good (>0.70) to very good (>0.81) for all scales. The criterion validity showed a strong correlation (0.76–0.78) between the numerical rating scale and VAS versus UCAPS and CPS, and between UCAPS and CPS (0.76). The UCAPS and CPS were responsive; all items and total scores increased after surgery. Both scales were specific (81–85%) and sensitive (82–87%). The cut-off point for rescue analgesia was >4 for UCAPS and >3 for CPS. Conclusions. The UCAPS and CPS are valid and reliable to assess postoperative pain in *Bos taurus* and *Bos indicus* bulls.

## 1. Introduction

Pain in bovines is often neglected [1]. The myth that farm animals suffer less pain than companion animals or that they have a more economical than effective value may explain why they receive fewer analgesics than other domestic animals [2,3,4]. Pain assessment is crucial to recognise and quantify pain and to ensure the welfare of farm animals [5].

Pain behaviours vary according to species. In cattle, pain may be masked or difficult to recognize. They have stoic and prey behaviours to avoid demonstrating vulnerability [1]. Therefore, a species-specific valid and reliable pain assessment instrument aids to identify and quantify pain to guide clinical decisions for providing analgesia [6,7,8,9].

Although there are several instruments that have been used for pain assessment in dairy and beef cattle, such as the UNESP-Botucatu cattle pain scale (UCAPS) [10], the cow pain scale (CPS) [11], the ‘posture scoring system’ [12], the ‘multidimensional pain scoring system’ [13], the ‘visual analog scale’ [14], the ‘veterinarian pain scale’ [15] and the ‘technician pain scale’ [15], only a few encompass robust validation criteria (i.e., content, criterion, and construct validity, intra- and inter-rater reliability, internal consistency, among others) [5,16].

After validity and reliability [5,8,16,17], the instruments require clinical validation to assess their application in clinical practice [18] and cross-cultural validation to be used in different languages and cultures. The latter has been performed for UCAPS in Portuguese and Italian [6].

*Bos taurus* and *Bos indicus* have endocrine/physiological differences, particularly related to reproduction [19,20,21] and behaviour (i.e., differences in vocalization) [9,22]. Therefore, it is essential that pain assessment instruments undergo breed- and species-specific validation. In fact, the UCAPS was developed for *Bos indicus* (Nelore) and the CPS for *Bos taurus* (Danish Holstein and Danish Holstein Friesian cattle). To the authors’ knowledge, there are no studies investigating the differences in pain assessment between these two species.

The aim of this study was to investigate the validity and reliability of the UCAPS [10] and the CPS [11] in *Bos taurus* (Angus) and *Bos indicus* (Nelore) bulls undergoing testicular warming followed by orchiectomy under general anaesthesia in the veterinary hospital scenario. The first hypothesis is that both scales would be valid and reliable to assess pain in both species. The second hypothesis is that pain scores would be similar between *Bos taurus* (Angus) and *Bos indicus* (Nelore).

## 2. Materials and Methods

The study protocol was approved by the School of Veterinary Medicine and Animal Science (University of São Paulo State—UNESP) Ethical Committee for the Use of Animals in Research (Approval number, 0147/2018) and followed the recommendations of COSMIN [23,24] and ARRIVE [25] guidelines adapted to the experimental design. The bulls were part of another experiment investigating the influence of testicular warming followed by castration. Respecting the three R’s, the perioperative pain assessment observations and video recordings of these patients were used in the current clinical prospective study. Both pain scoring instruments [10,11] used in this research were published in open-access journals, under the Creative Commons license (for the UCAPS: http://creativecommons.org/publicdomain/zero/1.0/ and the CPS: http://creativecommons.org/licenses/by-nc-nd/4.0/).

### 2.1. Procedure, Time-Points, and Video Recording

Twelve *Bos indicus*, Nelore breed, and nine *Bos taurus*, Angus breed, aged 19 to 24 months old, with a body weight of 250 to 450 kg (Nelore 451 kg ± 41 kg; Angus 264 kg ± 24 kg; mean ± SD) and considered healthy according to clinical and laboratory examination were included. The bulls were purchased from two private farms, one for each breed, transported and maintained separately in two groups (Nelore and Angus) at the Experimental Farm Lageado-FMVZ/UNESP. They were housed outdoors in two separate paddocks, fed with hay and grain and had *ad libitum* access to water. They were acclimatized to this site for one month before the start of the experiment. After this period, they were transported to the FMVZ/UNESP veterinary hospital in groups of three to four animals per week, where they were maintained under similar conditions receiving the same food and water *ad libitum*. The animals had a varied acclimatization time at the FMVZ/UNESP veterinary hospital, according to the order and date of the procedure for each animal. The first animal of the week had the shortest acclimatization time (2 h to 12 h) before being separated for fasting, and the other two animals of each week had a longer acclimatization time (24 h to 72 h). The experiments took place from 18 March to 29 May 2019. After the end of the experiments, the animals were kept at the Experimental Farm Lageado-FMVZ/UNESP for two months for fattening and then sent for humane slaughter.

At the FMVZ/UNESP veterinary hospital, each animal was individually fasted for water and food for 24 and 48 h, respectively, before the procedure. The animals were sedated with xylazine (0.05 mg/kg, Xilazin, Syntec do Brasil Ltd.a, Santana do Parnaíba, SP, Brazil) intravenously (IV). Five minutes later, a 14 g Teflon catheter (Nipro Medical Ltd.a, Sorocaba, SP, Brazil) was aseptically placed in the jugular vein, and anaesthesia was induced with ketamine (2.5 mg/kg, Dopalen, Ceva Saúde Animal Ltd.a, Paulínea, SP, Brazil) and diazepam (0.05 mg/kg, Compaz, Cristália, São Paulo, SP, Brazil) IV in the large animal induction room. Endotracheal intubation was performed with a 26 or 30 g silicon endotracheal tube. The patient was positioned in lateral recumbency on the surgical table, and anaesthesia was maintained with isoflurane (Isoforine, Cristália, São Paulo, SP, Brazil) in oxygen (15 L/min) using a large animal anaesthetic machine (Model 2800C, Mallard Medical, Redding, CA, USA), and the minimum alveolar concentration was monitored according to the end-tidal isoflurane concentrations and mean arterial pressure (60–90 mm Hg). Cardiorespiratory monitoring was performed using a multiparametric monitor (Cardiocap 5, GE-Datex, Finland) using electrocardiography, invasive arterial blood pressure, respiratory rate, capnography, and pulse oximetry. The anaesthetic depth was assessed via palpebral reflex, mandibular tone, and changes in sympathetic stimulation. Flunixin meglumine (1.1 mg/kg, Banamine, MSD Saúde Animal, Cruzeiro, SP, Brazil) was administered intramuscularly (IM), and xylazine (0.05 mg/kg diluted to a volume of 20 mL with saline 0.9%) was administered epidurally at the level of the sacrococcygeal intervertebral space [26]. Lactated ringer solution (10 mL/kg/h; Fresenius Kabi Brasil AS, Aquiraz, CE, Brazil) was administered IV during the procedure. Prophylactic antibiotic therapy (ceftiofur 1.1 mg/kg IM, Cef50, União Química Farmacêutica Nacional S/A, Embu-Guaçu, SP, Brazil) was given before surgical intervention.

The testicular artery and vein were isolated for blood sampling and blood flow assessment using an ultrasonic flowprobe (2SB1551; Transonic^®^ Flowprobe, Ithaca, NY, USA). The testicles underwent thermal warming with temperatures of 34, 37 and 40 °C for 45 min each as part of the experimental study. To modulate temperature, heat packs warmed to 45 °C or bags of ice were placed in contact with scrotal skin [27]. After the completion of the sampling and flow assessment, a bilateral scrotal incision was created for performing the orchiectomy. After the end of the surgical procedure, the animals were allowed to recover from anaesthesia. Post-operatively, the analgesic protocol consisted of morphine (administrated after time-point M2), dipyrone as rescue analgesia when needed (administered after M3) and flunixin meglumine (administrated at 24, 48 and 72 h after the end of surgery).

**Figure 1:**
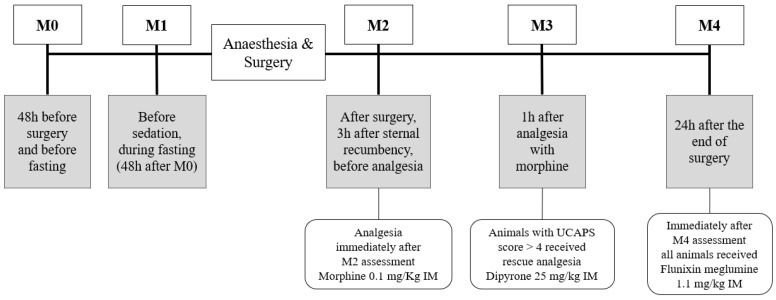
Timeline of the time-points used for the validation of the UNESP-Botucatu cattle pain scale (UCAPS) and the cow pain scale (CPS). Video recording was performed for 3 min at each time-point. Pain was assessed with the UCAPS, CPS, numerical rating scale (NRS) and visual analogue scale (VAS).

During the postoperative monitoring, the animals were kept in the same outdoor pens in groups of three or four. A camera (Canon PowerShot SX50 HS, Oita, Japan) was placed outside the outdoor pen, 1–2 m from the fence, using a camera tripod, and videos were recorded for a period of three minutes (without edition) at five time-points: M0—48 h before surgery and before fasting; M1—before sedation, during fasting (48 h after M0); M2—after surgery, three hours after animals were in sternal recumbency, before analgesia with morphine 0.1 mg/Kg IM; M3—one hour after morphine; when animals that showed a score >4 for UCAPS [10] received rescue analgesia with dipyrone 25 mg/kg IM; and M4—24 h after the end of surgery; when animals received flunixin meglumine at 1.1 mg/kg IM after the last evaluation (Figure 1).

### 2.2. Video Analysis

Two veterinarians with experience in pain assessment and unaware of the aforementioned time-points (A.R.O. and R.M.T.) performed the evaluations. Before starting the evaluations, training was performed using ten randomised videos of pre- and postoperative time-points of one Nelore and one Angus. These videos were excluded and were not part of phases 1 or 2.

A total of 95 videos of 3 min duration each resulting in 4.75 h of recordings were assessed twice (phases 1 and 2) by two evaluators, with a 30-day interval between phases. The two evaluators, unaware of the time-points, assessed each video with the sound on. They were allowed to watch the videos as many times as needed to complete the evaluations. The animals’ order and the five videos/time-points per animal were randomised for each phase of video analyses (http://www.randomization.com, accessed on 2 October 2019). The evaluators assessed five randomised videos/time-points for each animal per time. Once they finished each video, they completed the evaluations always in the same sequence as follows: (a) ‘Would you provide rescue analgesia according to your clinical experience?’ If yes, mark ‘1’, if no, mark ‘0’; (b) NRS and VAS; (c) UCAPS (Table S1) [10] and (d) CPS (for the item response to approach, they should complete it with a dot at the time-points the item did not happen) (Table S2) [11]. The evaluators were orientated to analyse the videos of a maximum of ten animals per week and for a maximum of one hour a day to avoid fatigue. The phase 1 video analysis occurred from 22 November to 22 December 2019. After a new randomization, the phase 2 video analysis occurred from 22 January to 22 February 2020 using the same recordings.

### 2.3. Statistical Methods

Statistical analyses were performed by a data scientist (P.H.E.T.) in R software with the RStudio integrated development environment (Version 4.1.0; 2021-06-29; RStudio, Inc., Boston, MA, USA) [28], using the data from the video analysis of phases 1 and 2 of both evaluators and all time-points for both breeds. The functions and packages used were presented in the format ‘package:function’ corresponding to the computer programming language in R. For all analyses, a *p* < 0.05 was considered for statistical significance. A minimum sample size of 11 subjects, with 0.80 of power and an alpha of 0.05 was calculated, based on Spearman correlation of *rho* = 0.764 between the UCAPS and CPS (http://biomath.info/power/, accessed on 10 January 2022). The Gaussian distribution was assessed according to the quantile–quantile and histogram plots, and the Shapiro–Wilk test that confirmed the data did not have a normal distribution. Hence, nonparametric tests were carried out for the analysis. Table 1 provides a detailed description of the statistical analysis.

## 3. Results

Ten Nelore (*Bos indicus*) and nine Angus (*Bos taurus*) bulls were included in the study. Two animals were excluded: one Nelore bull due to aggressive behaviour and another one due to post-operative radial nerve paralysis. The latter was treated with a different analgesic protocol and acupuncture and recovered promptly two days later. Four Angus and four Nelore bulls required rescue analgesia (dipyrone) at M3.

The mean duration of time from the induction of anaesthesia and the end of surgery was 5 h 43 ± 32 min. After the end of the surgery, the animals took 14 ± 5 min for extubating, 17 ± 7 min to spontaneously assume sternal recumbency and 38 ± 13 min to reach the quadrupedal position.

### 3.1. Distribution of Scores

**Figure 2:**
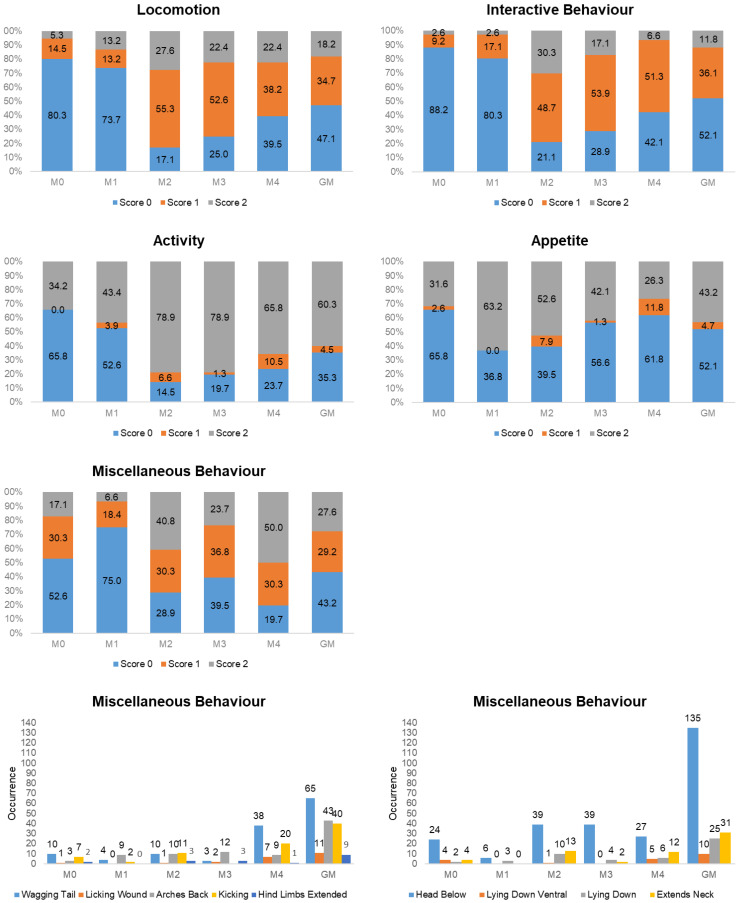
Occurrence frequency of each item’s score on the UNESP-Botucatu cattle pain scale (UCAPS). Percentage of the scores for each item and sum of occurrence for each item of the miscellaneous behaviours. Time-points: M0, 48 h before surgery and before fasting; M1, before sedation, during fasting (48 h after M0); M2, after surgery, three hours after animals were in sternal recumbency, followed by analgesia with morphine; M3, one hour after morphine; M4, 24 h after the end of surgery; GM, data of all time-points together (M0 + M1 + M2 + M3 + M4).

For the UCAPS, the score ‘0’ was predominantly observed at time-points M0, M1 and M4. Scores ‘1’ and ‘2’ were more frequently observed in M2 than in M3. The score ‘1’ for the item ‘activity’ and ‘appetite’ was not frequently observed. The three most frequent miscellaneous behaviours in M2 were ‘head below the line of spinal column’, ‘extends the neck and body forward when lying in ventral recumbency’ and ‘kicking/foot stamping’ (Figure 2).

**Figure 3:**
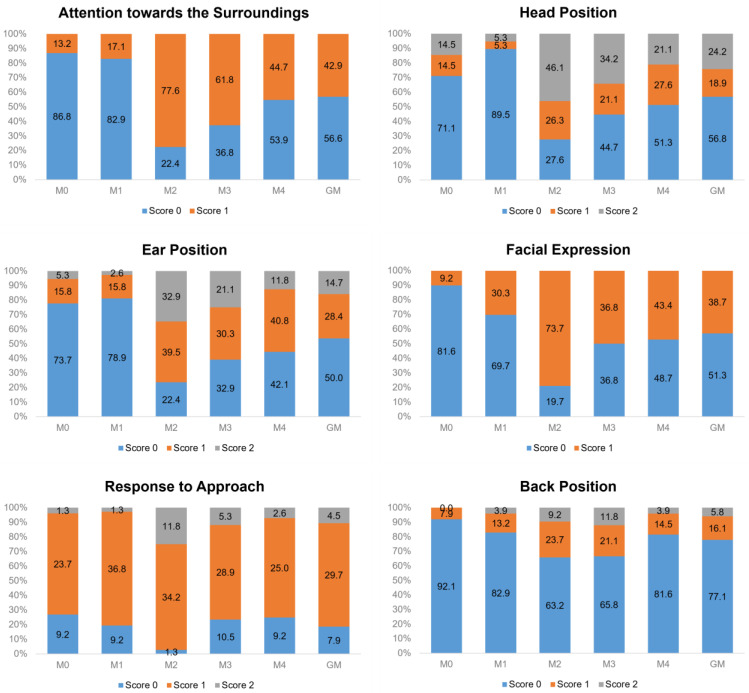
Occurrence frequency of each item’s score on the cow pain scale (CPS). Percentage of the scores for each item. Time-points: M0, 48 h before surgery and before fasting; M1, before sedation, during fasting (48 h after M0); M2, after surgery, three hours after animals were in sternal recumbency, followed by analgesia with morphine; M3, one hour after morphine; M4, 24 h after the end of surgery; GM, data of all time-points together (M0 + M1 + M2 + M3 + M4).

For the CPS, the score ‘2’ was not observed for ‘attention to surroundings’, ‘response to approach’ and ‘back position’ (Figure 3).

### 3.2. Multiple Association

**Figure 4:**
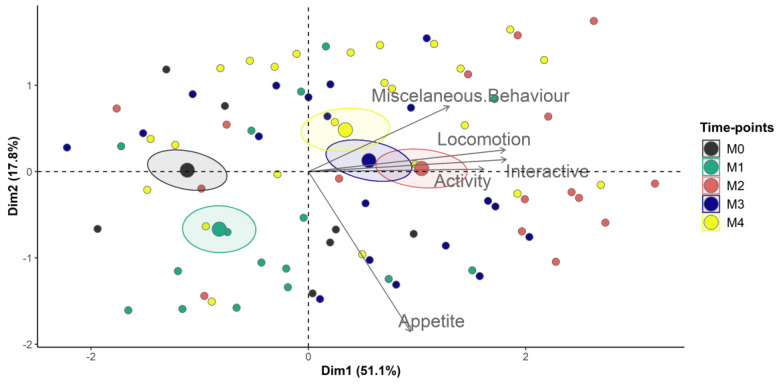
Biplot of the principal components analysis of the UNESP-Botucatu cattle pain scale (UCAPS). Confidence ellipses were built according to the time-points and pain scores. Time-points: M0, 48 h before surgery and before fasting; M1, before sedation, during fasting (48 h after M0); M2, after surgery, three hours after animals were in sternal recumbency, followed by analgesia with morphine; M3, one hour after morphine; M4, 24 h after the end of surgery. Ellipses were constructed according to the pain assessment time-points (M0 black, M1 green, M2 red, M3 blue and M4 yellow). The ellipse referring to the time when animals were suffering pain (M2) was positioned on the right side of the figure; on the opposite side are the ellipses corresponding to the time-points in which animals were pain-free (M0 and M1), where items showed lower scores. The time-point of moderate pain (M4) is positioned on the right side closer to the centre. All items on the scale are influenced by time-points of pain (M2 and M3) as their vectors are positioned in the direction of these ellipses and demonstrated higher pain scores.

**Figure 5:**
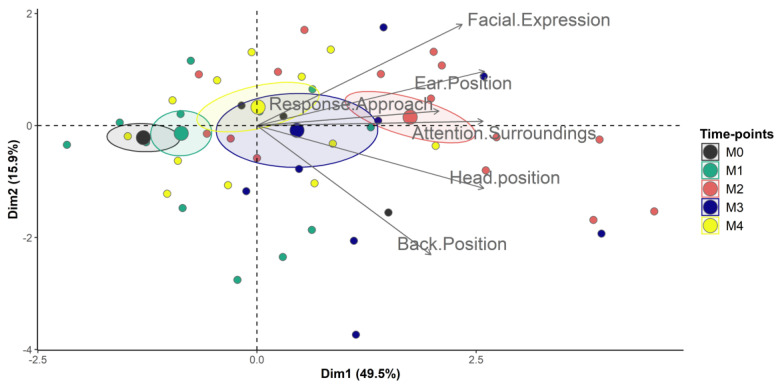
Biplot of the principal components analysis of the cow pain scale (CPS). Confidence ellipses were built according to the time-points and pain scores. Time-points: M0, 48 h before surgery and before fasting; M1, before sedation, during fasting (48 h after M0); M2, after surgery, three hours after animals were in sternal recumbency, followed by analgesia with morphine; M3, one hour after morphine; M4, 24 h after the end of surgery. Ellipses were constructed according to the pain assessment time-points (M0 black, M1 green, M2 red, M3 blue and M4 yellow). The ellipse referring to the time when animals were suffering pain (M2) was positioned on the right side of the figure; on the opposite side are the ellipses corresponding to the time-points in which animals were pain-free (M0 and M1), where items showed lower scores. The time-point of moderate pain (M4) is positioned on the right side closer to the centre. All items on the scale are influenced by time-points of pain (M2 and M3) as their vectors are positioned in the direction of these ellipses and demonstrated higher pain scores.

The Horn’s parallel analysis and the principal component of analysis (PCA) indicated that UCAPS and CPS are unidimensional (Table 2, Figure 4 and Figure 5).

### 3.3. Intra-Rater (Repeatability) and Inter-Rater (Reproducibility) Reliability

The repeatability of all scales was good for both evaluators (>0.75) (Table 3) and varied from very good to poor for the scale items. The reproducibility was good for all scales (≥0.73) (Table 4).

### 3.4. Concurrent Criterion Validity

The correlations between NRS and VAS versus UCAPS and CPS were strong (0.75–0.77), as well as between the UCAPS and CPS scales (0.76) (Table 5).

### 3.5. Responsiveness

**Figure 6:**
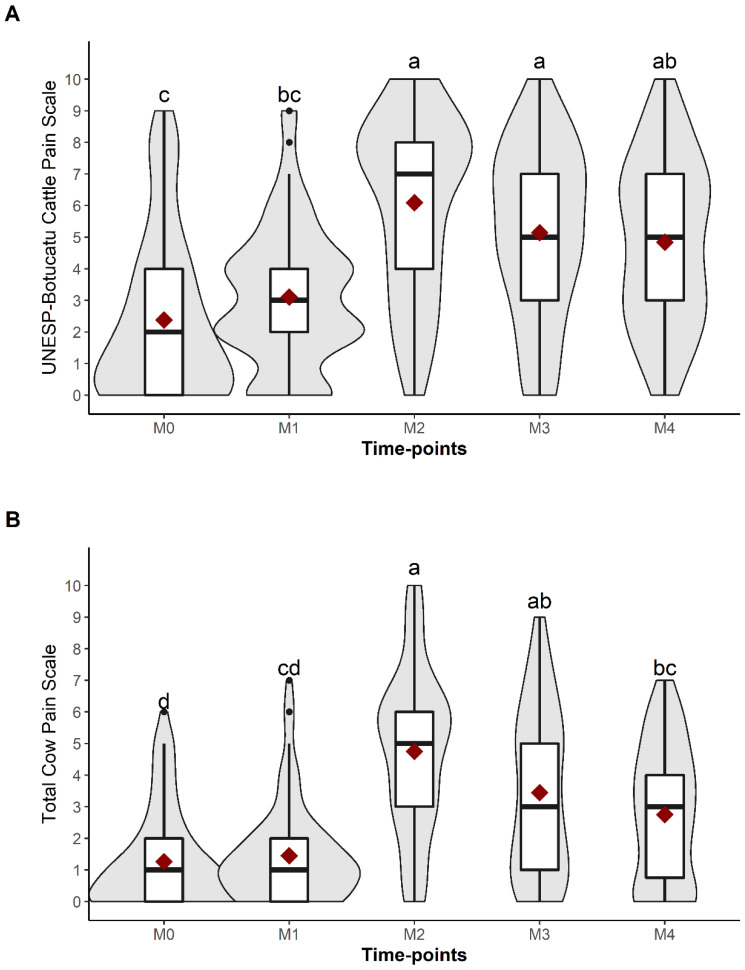
Violin and box plot of the UNESP-Botucatu cattle pain scale (UCAPS) and cow pain scale (CPS) over time-points. (**A**) UCAPS total score; (**B**) CPS total score. The violin contour represents the dispersion data density together (both evaluators’ data), with the wider contour representing greater data density; the lower and upper bounds of the box, respectively, represent the first and third quartiles of data; the horizontal line plus space inside the box indicate the median; the red diamond indicates the average of each piece of time-point data separately; black circles indicate outliers. Different lowercase letters indicate statistical differences over the time-points (a > b > c > d); multiple comparisons were conducted by a linear mixed model with post-test corrected by Bonferroni procedure (*p* < 0.05). Time-points: M0, 48 h before surgery and before fasting; M1, before sedation, during fasting (48 h after M0); M2, after surgery, three hours after animals were in sternal recumbency, followed by analgesia with morphine; M3, one hour after morphine; M4, 24 h after the end of surgery.

**Figure 7:**
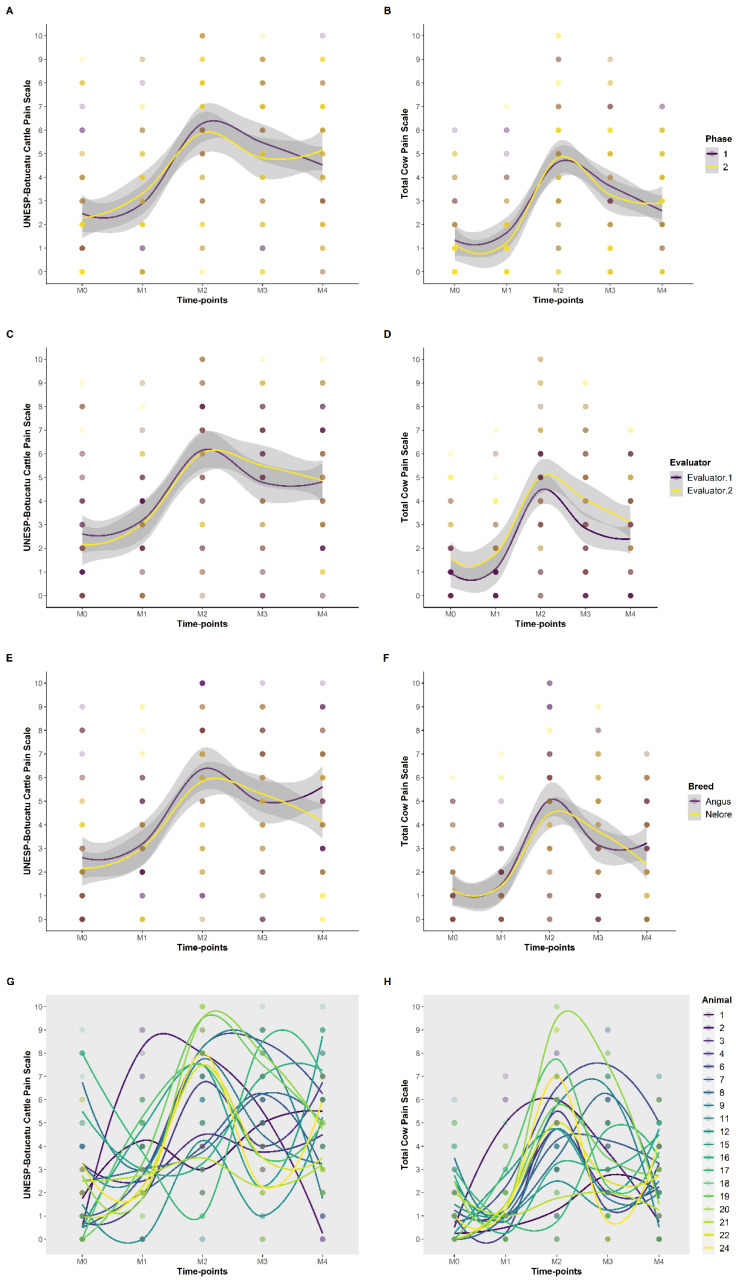
Smooth line to total sum of the UNESP-Botucatu cattle pain scale (UCAPS) and cow pain scale (CPS) over the time-points for each phase, evaluator, breed and animal. (**A**) UCAPS, for phases 1 and 2. (**B**) CPS, for phases 1 and 2. (**C**) UCAPS, for evaluators 1 and 2. (**D**) CPS, for evaluators 1 and 2. (**E**) UCAPS, for Angus and Nelore. (**F**) CPS, for Angus and Nelore. (**G**) UCAPS, for each animal individually. (**H**) CPS, for each animal individually. The smooth lines were created automatically by the loess method; the grey area represents the standard error of the smooth line. Time-points: M0, 48 h before surgery and before fasting; M1, before sedation, during fasting (48 h after M0); M2, after surgery, three hours after animals were in sternal recumbency, followed by analgesia with morphine; M3, one hour after morphine; M4, 24 h after the end of surgery.

The total scores of UCAPS and CPS were significantly higher at M2 than at M0 and M1 (Table 6, Figure 6), demonstrating their responsiveness, even though there were no statistical differences between M2, M3 and M4 for the UCAPS total score, and between M2 and M3 for the CPS total score. ‘Phases’ (Figure 7A,B), ‘evaluators’ (Figure 7C,D) and ‘breed’ (Figure 7E,F) did not have a significant effect on the UCAPS total scores. ‘Evaluator’ significantly changed the CPS total scores. There was no influence of ‘breed’ and ‘evaluators’ (as fixed effects) in the UCAPS and CPS total scores or the time-points for the UCAPS. For the CPS, ‘time-points’ M3 and M4 affected the scores (as fixed effects) (Tables S3 and S4). There was individual variation for each animal when looking at the total sum of UCAPS and CPS over each time-point (Figure 7G,H), justifying the use of a mixed model which accounts for the individual effect.

The differences between the time-points for the total scores of UCAPS were M2/M3/M4 > M1 > M0; for the CPS and NRS M2/M3 > M4 > M1 > M0; and for VAS M2/M3/M4 > M1/M0 (Table 6, Figure 6). There was a significant difference, M2 > M1/M0, demonstrating the scale’s responsiveness, for the total score and all items of UCAPS and CPS, except for ‘appetite’ and ‘response to approach’, respectively.

There was no influence based on breed when the total scores of UCAPS, CPS, NRS and VAS were compared. However, Angus animals had higher scores for ‘miscellaneous behaviour’ and ‘back position’ (Table 6), as well as a two-fold increase for Angus compared to Nelore for the UCAPS sub-items ‘moves and arches the back when in standing posture’, ‘lying down with the head on/close to the ground’ and ‘extends the neck and body forward when lying in ventral recumbency’. Moreover, ‘phase’ as a fixed effect influenced the VAS total score, ‘evaluator’ influenced the NRS total score, CPS total score, and the items ‘locomotion’, ‘interactive behaviour’, ‘appetite’, ‘head position’, ‘ear position’ and ‘back position’ (Table 6).

### 3.6. Construct Validity

The scores of the postoperative time-point M2 were significantly higher when compared with the preoperative time-points M0 and M1 (Table 6), which characterizes the ability of the scales to measure pain. However, there was no statistical difference in the scores after analgesia (administration of morphine) for all scales (Table 6).

The results demonstrated that the UCAPS and CPS were responsive to changes in pain scores and discriminated against intense pain (M2), and CPS discriminated against moderate pain (M4). However, the scales were not responsive to analgesia (administration of morphine) (Table 6, Figure 6).

### 3.7. Internal Consistency

The Cronbach’s α coefficient was 0.72 and 0.79 for UCAPS and CPS, indicating good and very good internal consistency, respectively. Internal consistency for ‘appetite’ (UCAPS) was higher than the total scores, indicating that these items provide less contribution to the total scores than the others (Table 7). McDonald’s omega coefficient was 0.79 and 0.88 for the full-scale UCAPS and CPS, indicating acceptable and strong internal consistency, respectively (Table 7).

### 3.8. Item-Total Correlation

Except for ‘appetite’ (0.28) for UCAPS, the item-total correlation coefficient of items from UCAPS and CPS ranged from 0.39 to 0.69. Therefore, all items were accepted as they were between 0.3 and 0.7, except for ‘appetite’ [16] (Table 7).

### 3.9. Specificity and Sensibility

Both scales showed specificity and sensitivity. Most items were specific for both scales, except for ‘appetite’ and ‘miscellaneous behaviour’ for the UCAPS, and ‘response to approach’ for the CPS. Most items presented moderate sensitivity, except ‘appetite’ (UCAPS) and ‘back position’ (CPS) (Table 8).

### 3.10. Determination of a Cut-Off Point for the Administration of Rescue Analgesia

The receiver operating characteristic (ROC) curve determined the cut-off point for diagnosing pain and recommending analgesia. The Youden index was ≥5 out of 10 for the UCAPS and ≥3 out of 10 for the CPS (Table 9). The resampling confidence interval >0.90 for the Youden index was between 4.6–5.5 and 2.55–3.1 for the UCAPS and CPS, respectively. Based on the resampling result, the diagnostic uncertainty zone scores were 5, and 3, for UCAPS and CPS, respectively. Scores < 5 and <3 indicate pain-free cattle (true negative) and >5 and >3 indicate cattle suffering pain (true positive) for UCAPS and CPS, respectively.

**Figure 8:**
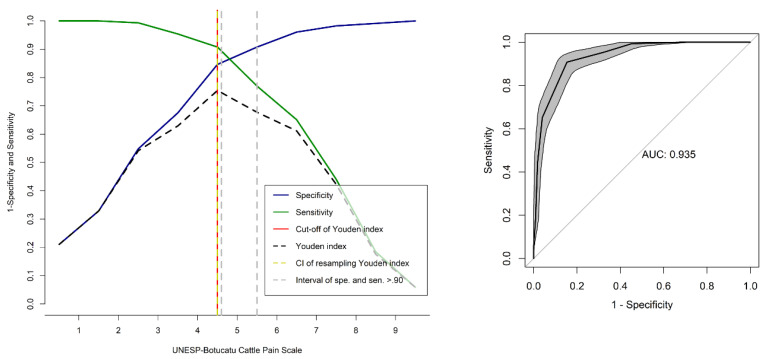
Two-graph ROC curve with the diagnostic uncertainty zone and ROC curve with AUC for the UNESP-Botucatu cattle pain scale (UCAPS). ROC (receiver operating characteristic) curve with a 95% confidence interval (CI) calculated from 1001 replications and area under the curve (AUC) [16]. The interpretation of an AUC ≥ 0.95 indicates a high discriminatory ability. Two-graph ROC curve, CI of 1001 replications, and of sensitivity and specificity >0.90 were applied to estimate the diagnostic uncertainty zone of the cut-off point of all evaluators, according to the Youden index for the UCAPS [42,43]. The diagnostic uncertainty zone was 5–6; <4 indicates pain-free animals (true negative) and >5 indicates animals suffering pain (true positive). The Youden index was >4, representative of the cut-off point for the indication of rescue analgesia.

**Figure 9:**
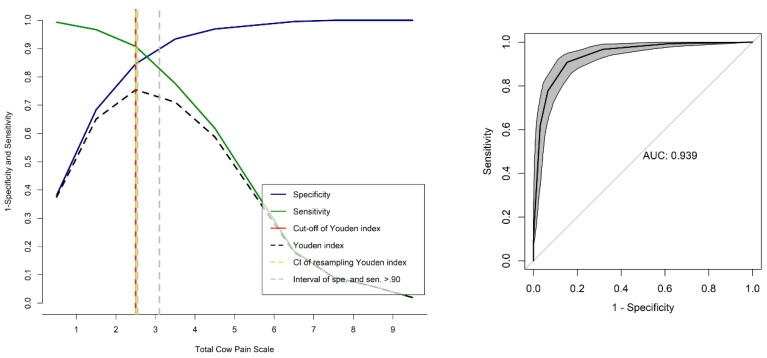
Two-graph ROC curve with the diagnostic uncertainty zone, and ROC curve with AUC for the cow pain scale (CPS). ROC (receiver operating characteristic) curve with a 95% confidence interval (CI) calculated from 1001 replications and area under the curve (AUC) [16]. Interpretation of AUC ≥ 0.95 indicates high discriminatory ability. Two-graph ROC curve, CI of 1001 replications, and of sensitivity and specificity >0.90 applied to estimate the diagnostic uncertainty zone of the cut-off point of all evaluators, according to the Youden index for the UCAPS [42,43]. The diagnostic uncertainty zone was 3–4; <3 indicates pain-free animals (true negative) and >4 indicates animals suffering pain (true positive). The Youden index was >3, representative of the cut-off point for the indication of rescue analgesia.

The area under the curve (AUC) was 0.93 for both UCAPS and CPS, indicating that both scales have excellent discrimination between painful and non-painful individuals (Figure 8 and Figure 9). For the unidimensional scales, the cut-off points for rescue analgesia defined by the ROC curve and the Youden index were ≥4 for NRS, and ≥32 for VAS (Table 9), and the AUCs for NRS and VAS were 0.99 and 0.99, respectively.

## 4. Discussion

This study showed that the UCAPS and CPS are valid and reliable pain-scoring instruments to be used in *Bos taurus* (Angus) and *Bos indicus* (Nelore) bulls undergoing general anaesthesia and orchiectomy, confirming the first hypothesis of the study. Both scales were capable of assessing and differentiating pain-free individuals from those experiencing pain, both scales can support farm animal veterinarians to assess pain, and the results provide robust evidence that cattle feel pain after castration [46,47]. The second hypothesis was also confirmed, as there were no significant differences in the pain scores between the two species of cattle.

The lack of differences in the pain scores between the cattle species suggests that they express pain similarly and may disprove that, anecdotally, *Bos indicus* is ‘tougher’ than *Bos taurus*, and that the first show or ‘feel’ less pain than the second species. Previous studies demonstrated physiological differences between the two species [19,20,21], but this was the first study comparing pain behaviour scores between the two breeds. Future research should compare female versus male individuals to investigate if the scales are applicable to both sexes and diverse types of pain.

The distribution of scores with the UCAPS and CPS followed an expected pattern similar to previous studies involving the validation of a pain scale in sheep [8] and pigs [17]. In general, individual item scores were predominantly zero before surgery, increased to the highest values after surgery, decreased after the administration of analgesia (morphine) and decreased again 24 h after surgery.

The principal component analysis (PCA) tests the dimensionality of an instrument [16], which is an important part of validity [18,48]. For example, the PCA and the Horn’s parallel analysis identify the number of dimensions or domains of a scoring instrument [31,49] and are prerequisites for the interpretation of internal consistency by McDonald’s omega [16,18]. Both UCAPS and CPS are unidimensional like the pain scales in pigs [17] and sheep [8]. Because pain is a multidimensional experience [50,51], the UCAPS and CPS may be considered multidimensional instruments in biological terms, because they include components of pain intensity, qualitative and temporal aspects, physiological (e.g., ‘appetite’ for UCAPS), motor (e.g., ‘locomotion’ and ‘activity’ for UCAPS), sensory (‘miscellaneous behaviours’ for UCAPS; ‘back position’ for CPS) and emotional and cognitive attributes (e.g., ‘interactive behaviour’ for UCAPS; ‘attention towards the surroundings’, ‘head position’, ‘ear position’, ‘facial expression’ and ‘response to approach’ for CPS).

Both pain scoring instruments tested in this study are reliable when using two evaluators with experience in pain assessment. The intra-rater reliability of UCAPS and CPS was superior to previous pain scoring instruments developed for cattle, the Posture Scoring System (PSS) [12] and the Pain Assessment Based on Facial Expression [52], similar to the UNESP-Botucatu composite scale to assess acute postoperative abdominal pain in sheep (USAPS) [8] and inferior to the UNESP-Botucatu pig composite pain scale (UPAPS) [17]. The inter-rater reliability of UCAPS and CPS was inferior to UPAPS [17] and superior to USAPS [8], the PSS [12], and the visual analogue composite scale [14]. These outcomes may be a consequence of different criteria used and the different number of evaluators of other studies.

The criterion validity demonstrates the degree to which the scores are an adequate reflection of a ‘gold standard’ or other previously validated method for measuring the same construct [53]. The closest ‘gold standard’ instrument for the bovine species is the UCAPS itself [10]. On the other hand, another approach previously used to validate other animal pain scales involves the comparison of the testing instrument against unidimensional scales, such as NRS, simple descriptive scale (SDS), and VAS [6,8,10,17,34,54,55,56,57]. The strong correlation among NRS, VAS, UCAPS and CPS confirmed the criterion validity, like in these previous studies in other animal species and in the original UCAPS study [10].

Construct validity is the degree to which the score of an instrument corresponds to the target measurement [53], for instance, by discriminating pain vs. pain-free states. Responsiveness, which can be considered a ‘longitudinal validity’ [58], is the ability of an instrument to measure changes in the construct to be measured (e.g., pain in this case) by increasing the scores after painful events and/or decreasing the scores after the administration of analgesia [16,59]. The UCAPS and CPS identified the differences in scores over time, after surgery and baseline, similar to several other pain scales including the original UCAPS study [10], and the sheep [8], pig [17], horse [54,56], donkey [57] and cat [34]. However, none of the instruments were responsive to the analgesia (the administration of morphine), and only the CPS decreased the score by 24 h following surgery. This result might have happened because the analgesia protocol (morphine 0.1 mg/kg) at M2 was insufficient, and indeed a total of eight animals required rescue analgesia (dipyrone) at M3. It is not known how residual anaesthesia or sedation may have influenced these results. The mitigation of pain in farm animals is a challenge to livestock caretakers, and dosage regimens for opioid analgesics have not been well established in these species [60]. Another possible explanation relates to the more intense nociceptive stimulus used in this study, due to the application of thermal warming leading to more inflammation compared to the original study [10] that used castration under sedation with xylazine and local anaesthesia. Other studies showed that animal pain assessment could be influenced by gender [61,62,63,64] or by how experienced the evaluator is [17,65]. Furthermore, a study with laboratory animals showed that training improved pain recognition [66]. A recent study in cats demonstrated that female evaluators assigned higher scores than male ones [62], and another study showed that experience might improve reliability during pain assessment [65]. The impact of gender and the evaluator’s experience in cattle is still to be investigated and it is not clear how this may have influenced our results using only two female evaluators.

An interesting finding was that fasting did not influence the behaviours included in UCAPS and CPS. This result suggests that both scales may be used to assess pain regardless of fasting conditions. Preoperative fasting could have influenced pain scores because water and food deprivation can cause behavioural and physiological changes [67] and discomfort and stress [68]. The UCAPS total score was not affected by any fixed effect variable, and although ‘phase’ influenced the VAS, and ‘evaluator’ influenced the NRS and CPS total scores, ‘breed’ did not affect the responsiveness of the total score of the scales, neither most of the items, confirming that all scales may be used in both breeds. Additionally, observation for 3 min is apparently sufficient for pain assessment using UCAPS and CPS in cattle, showing that these instruments can be easily included in clinical routines.

The internal consistency confirms that the items represent well the changes in pain intensity, can be added to the scale total score and are related to the overall pain assessment [8,34]. Our results were slightly inferior to previous ones in different species [8,10,17,69]. This finding might have happened because the conditions of this study were different from the original validation study [10]. Another potential factor to consider is the importance of items. For instance, ‘appetite’ (UCAPS), ‘back position’ and ‘response to approach’ (CPS) minimally contributed to the scale total scores. Except for ‘appetite’, all items from UCAPS and CPS exhibited acceptable item-total correlation as in pigs [17] and sheep [8], ensuring the relevance and homogeneity of the items within the scales.

Both UCAPS and CPS identified most accurately pain cases after surgery and most truly painless cases before surgery, therefore confirming sensitivity and specificity, respectively. Again, like for item-total and internal consistency, ‘appetite’ and ‘miscellaneous behaviour’ for UCAPS and ‘response to approach’ for CPS were not specific, and ‘appetite’ (UCAPS) and ‘back position’ (CPS) were not sensitive, showing that these items may contribute little to the scales, suggesting that a refinement process might be required.

The ROC curve estimated the optimal cut-off point to diagnose pain and recommend analgesia [41] with high sensitivity and specificity and excellent discriminatory capacity for diagnosis accuracy, like in previous studies [8,10,17,69]. The cut-off point for the administration of analgesics guides decision-making in the clinical setting and provides information about the effectiveness and duration of analgesics [34]. In the current study, the same cut-off points were found for UCAPS and CPS as in the original studies [10,11]. The determination of the diagnostic uncertainty zone improves certainty in correctly defining pain-free (true negatives) and pain states (true positives) [17]. This study was the first to perform this calculation in cattle.

This study has limitations. The in-person researcher who recorded the videos was one of the evaluators, and although she was unaware of the time-points and animals, this could have still generated expectation bias [70]. However, we believe that this bias was mitigated by the six-month interval between the video recordings and evaluations. Another limitation is the lack of randomisation for the assessment order of the pain scales, yet the NRS and VAS being assessed first might have influenced the scores of each other and UCAPS and CPS [8,10,17,56,69]. Another limitation was not assessing the animals which received rescue analgesia (dipyrone) after the time-point M3, to investigate the effectiveness of analgesia administration [71]. Thus, this should be implemented in future studies.

The number of evaluators was small. A recent study suggested that three evaluators would be ideal [5], suggesting that future studies should be performed with more evaluators. Another limitation is that this study only evaluated male individuals, and future research should be carried out to understand if these results can be extrapolated to procedures involving cows. Finally, other types of pain including individuals of different ages, sexes, clinical conditions, and painful states should be used for further validation of the UCAPS and CPS, testing the psychometrical properties of these scales in other languages and cultures [61], and performing statistical weighting of each behaviour to define their importance like in sheep [72].

## 5. Conclusions

The UCAPS and CPS are valid, sensitive, specific, consistent, and reliable instruments to assess postoperative pain in *Bos taurus* (Angus) and *Bos indicus* (Nelore) bulls, without differences in pain scores between the breeds. Their cut-off points indicate decision-making for providing rescue analgesia in the clinical setting.

## Figures and Tables

**Table 1:** Statistical methods used for psychometrical testing of the UNESP-Botucatu cattle pain scale (UCAPS) and the cow pain scale (CPS) [8,17,29,30].

Type of Analysis	Description	Statistical Test
Distribution of scores	1-Frequency distribution of the presence of the scores ‘0′, ‘1′ and ‘2′ of each item of the UCAPS and CPS at each time-point and all time-points grouped (MG).	Descriptive analysis.
Multiple association	The multiple association of the scale items was analysed at all time-points grouped using principal component analysis in order to define the number of dimensions (components) determined by different variables that establish the scale extension.	Principal component analysis (stats::princomp and factoextra::get_pca_var) was performed. Horn’s parallel analysis [31] (psych::fa.parallel) was performed to determine the optimal number of principal components to be retained. Significant associations were considered when the loading value was ≥0.50 or ≤−0.50. For the biplot, confidence ellipses were produced with 95% significance levels to show the density of assessments at each time point.
Intra-rater reliability	Repeatability—the level of agreement between each rater with themselves was estimated by comparing the two phases of assessment, using the scores of each item and the total sum of all scales and the need for rescue analgesia.	For the scores of the items of the UCAPS, CPS and NRS, the weighted kappa coefficient (kw) was used; the disagreements were weighted according to their distance to the square of perfect agreement (psych::cohen.kappa). The 95% confidence interval (CI) kw based on 1001 replications by the bootstrap method was estimated (boot::boot.ci). For the sum of the UCAPS and CPS, the intraclass correlation coefficient (ICC; irr::icc) was used by applying the two-way mixed and alpha model, type consistency multiple. For VAS, the intraclass correlation coefficient (ICC; irr::icc) was used by applying the two-way mixed and alpha model, and type absolute agreement. Evaluators/measurements and their 95% CI based on 1001 replications were calculated by the bootstrap method (boot::boot.ci). Interpretation of kw and ICC: very good 0.81–1.0; good: 0.61–0.80; moderate: 0.41–0.60; reasonable: 0.21–0.4; poor < 0.2 [16,32,33,34].
Inter-rater reliability	Reproducibility—the level of agreement between the two evaluators using the scores for the item, and the total sum of all scales was assessed.	
Criterion validity	(1) Concurrent criterion validity (comparison with a validated instrument)—the total score of UCAPS and CPS was correlated with the VAS and NRS and between each other.	Spearman rank correlation coefficient (rs; Hmisc::rcorr). Interpretation of the degree of correlation rs (*p* < 0.05): very weak < 0.19; weak 0.2–0.39; moderate 0.4–0.59; strong 0.6–0.79; very strong 0.8–1 [35]
	(2) Concurrent criterion validity—the agreement between evaluator 1 vs. evaluator 2 (reproducibility).	See previous description for inter-rater reliability.
	(3) Predictive criterion validity was assessed by the number of animals that should receive rescue analgesia according to the Youden index (described below) in the time-point of greatest pain (M2).	Descriptive analysis.
Responsiveness	Responsiveness—the scores of each item, and total sum of the scale were compared over time (M0 vs. M1 vs. M2 vs. M3 vs. M4). Interpretation: differences in scores are expected to occur as follows: M2 > M4 > M3 > M1 = M0.	The residual models (stats::residuals) for all the dependent variables did not fit into Gaussian distribution according to the quantile–quantile and histogram plots (stats::qqnorm and lattice::histogram); thus, generalized mixed linear models (lme4::glmer) were applied. For the dichotomous variables based on miscellaneous behaviours, logistic regression analysis (stats::glm) was applied. Time-points, evaluators, and breed were included as fixed effects and the animals as random effects in all the models. Tukey test was used as a post hoc test [8].
Construct validity	Construct validity was determined by four methods: 1. Three hypotheses’ tests: (i) if the scale measures pain, the score after surgery (M2) should be higher than the preoperative score (M0 and M1); (ii) pain scores should decrease after analgesia (M3 < M2) and (iii) 24 h pain scores should be between preoperative (M0 and M1) and M2 assessments.	See Responsiveness.
	2. Known-groups validity: Pain-free animals (M0 and M1) should have lower pain scores than animals suffering pain (M2).	Mann–Whitney test (stats::wilcox.test) [36]
	3. Internal relationships among items.	See internal consistency, item-total correlation, and principal component analysis.
	4. Relationships to scores of other instruments.	See criterion validity.
Internal consistency	The consistency (interrelation) of the scores of each item of the scale was estimated.	Cronbach’s alpha coefficient (α; psy::cronbach) and McDonald’s omega coefficient [37] were performed (ω; psych::omega). Cronbach’s alpha coefficient interpretation: 0.60–0.64, minimally acceptable; 0.65–0.69 acceptable; 0.70–0.74 good; 0.75–0.80 very good; and >0.80 excellent [38,39]. McDonald’s omega coefficient interpretation: 0.65–0.80, acceptable; >0.80 strong reliability evidence [37].
Item-total correlation	The correlation of each item of the scale after excluding the evaluated item was estimated to analyse homogeneity, the inflationary items, and the relevance of each item of the scale.	Spearman rank correlation coefficient (r2; Hmisc::rcorr). Interpretation of item-total correlation r: suitable values 0.3–0.7 [16]
Specificity and Sensitivity	Specificity: based on true negatives (TN)—animals without pain (score 0) at M0. Sensitivity: based on true positives (TP)—animals with pain (scores 1 or 2) ate M2. The scores of each item of the scales were transformed into dichotomous (score ‘0’—absence of pain expression behaviour for a given item; scores ‘1’, and ‘2’—presence of pain). For the total score of the scale, the percentage of animals that had score < 4 at M0 and >4 (cut-off point) at M2 was considered to be specific and sensitive, respectively.	$\mathrm{Sp}\;M0=\frac{\mathrm{TN}}{\mathrm{Total}\;\mathrm{number}}$ and $S\;M2=\frac{\mathrm{TP}}{\mathrm{Total}\;\mathrm{number}}$ Specificity, sensitivity and its 95% CI were calculated according to the bootstrap method described below (epiR::epi.tests). Interpretation: excellent 95–100%; good 85–94.9%; moderate 70–84.9%; not specific or not sensitive <70% [40]
Optimum cut-off point	Time-points (M0 and M1) of pain-free animals and one of the postoperative time-points (M2) were used to determine the optimal cut-off point. Data associated with the suggestion of rescue analgesia according to clinical experience were used as the true value and the total score of the scales as a predictive value to build a ROC curve. The cut-off point for rescue analgesia was determined based on the Youden index and its diagnostic uncertainty zone using all moments of pain assessment on the scales. The AUC was calculated and indicates the discriminatory capacity of the test.	Cut-off point was based on the Youden index (YI = [Sensitivity + Specificity] − 1), which determines the highest concurrent sensitivity (true positives) and specificity (true negatives) value from the Receiver Operating Characteristic (ROC) curve (pROC::roc and pROC::ci.sp’). The discriminatory capacity of the scale was determined by the area under the curve (AUC). AUC values above 0.90 represent the high accuracy discriminatory capacity of the scale [41]. In addition, the 95% confidence interval (CI) was calculated from the Youden index by replicating the original ROC curve 1001 times according to the bootstrap method (pROC::ci.coords and pROC::ci.auc). The diagnostic uncertainty zone was determined by two methods: (1) calculating the 95% confidence interval (CI) by replicating the original ROC curve 1001 times according to the bootstrap method and (2) calculating the sensitivity and specificity value >0.90. The lowest and highest values of these two methods among all evaluators were assumed to be the diagnostic uncertainty zone [42,43].

Scales: NRS, numerical rating scale; VAS, visual analogue scale. Time-points: M0, 48 h before surgery and before fasting; M1, before sedation, during fasting (48 h after M0); M2, after surgery, three hours after animals were in sternal recumbency, followed by analgesia with morphine 0.1 mg/kg IM; M3, one hour after morphine; when animals that showed a score >4 for UCAPS [10] received dipyrone 25 mg/kg IM; M4, 24 h after the end of surgery; when animals received flunixin meglumine at 1.1 mg/kg IM after the last evaluation.

**Table 2:** Loading values, eigenvalues, and variance of the UNESP-Botucatu cattle pain scale (UCAPS) and cow pain scale (CPS) items based on principal components analysis.

Variables	Dimension 1	Dimension 2
	Loading Value	Loading Value
**UCAPS items**		
Locomotion	**0.84**	0.12
Interactive behaviour	**0.85**	0.07
Activity	**0.75**	0.01
Appetite	0.44	**−0.86**
Miscellaneous behaviours	**0.60**	0.35
**Eigenvalue**	**2.55**	0.35
**Variance**	**51.0**	**68.8**
**CPS items**		
Attention towards surroundings	**0.76**	0.02
Head position	**0.77**	−0.33
Ear position	**0.77**	0.29
Facial expression	**0.69**	**0.54**
Response approach	**0.62**	0.08
Back position	**0.59**	**−0.68**
**Eigenvalue**	**2.97**	0.95
**Variance**	49.5	15.9

The structure was determined considering items with a load value ≥0.50 or ≤−0.50 (in bold) [31,44].

**Table 3:** Intra-rater reliability of the UNESP-Botucatu cattle pain scale (UCAPS), cow pain scale (CPS), unidimensional scales and analgesia rescue indication in the perioperative period of cattle submitted to castration.

	Evaluator 1			Evaluator 2		
Variables	Weighed Kappa	Lower	Upper	Weighed Kappa	Lower	Upper
Rescue Analgesia	0.69	0.54	0.82	0.56	0.38	0.72
NRS	0.84	0.77	0.90	0.75	0.64	0.83
	ICC	CI Lower	CI Upper	ICC	CI Lower	CI Upper
Total Score UCAPS	0.89	0.84	0.92	0.79	0.69	0.86
Total Score CPS	0.86	0.79	0.90	0.78	0.67	0.85
VAS	0.91	0.86	0.94	0.82	0.73	0.88

Scales: NRS, numerical rating scale; VAS, visual analogue scale. Statistical tests: ICC, Intra-class correlation coefficient; CI, Confidence interval 95%. Interpretation of reliability: very good 0.81–1.0; good 0.61–0.80; moderate 0.41–0.60; reasonable 0.21–0.4; poor < 0.2 [16,34,45]. ICC model: alpha, two-way mixed; type: consistency for UCAPS and CPS, and absolute agreement for VAS. Rescue analgesia was indicated based on the evaluator’s experience answering a question before assessing the pain scales ‘Do you think it is necessary to provide rescue analgesia?’ yes (1) or no (0).

**Table 4:** Inter-rater reliability of the total score of UNESP-Botucatu cattle pain scale (UCAPS), cow pain scale (CPS), unidimensional scales and rescue analgesia indication in the perioperative period of cattle submitted to castration.

	Evaluator 1 Versus Evaluator 2		
Variables	Weighed Kappa	Lower	Upper
Rescue Analgesia	0.47	0.34	0.59
NRS	0.73	0.65	0.79
	ICC	CI Lower	CI Upper
Total Score UCAPS	0.80	0.73	0.85
Total Score CPS	0.74	0.65	0.80
VAS	0.82	0.76	0.86

Scales: NRS, numerical rating scale; VAS, visual analogue scale. Statistical tests: ICC, Intraclass correlation coefficient; CI, Confidence interval 95%. Interpretation of reliability: very good 0.81–1.0; good 0.61–0.80; moderate 0.41–0.60; reasonable 0.21–0.4; poor < 0.2 [16,34,45] ICC model: alpha, two-way mixed; type: consistency for UCAPS and CPS and absolute agreement for VAS. Rescue analgesia was indicated based on the evaluator’s experience answering a question before assessing the pain scales ‘Do you think it is necessary to provide rescue analgesia?’ yes (1) or no (0).

**Table 5:** Spearman’s correlation matrix between the total scores of the UNESP-Botucatu cattle pain scale (UCAPS), cow pain scale (CPS) and unidimensional scales to assess pain in cattle.

	Spearman Correlation (*rho*)		
Scales	UCAPS	CPS	VAS
UCAPS	-	-	-
CPS	0.76	-	-
VAS	0.75	0.77	-
NRS	0.76	0.77	0.97

Scales: VAS, visual analogue scale; NRS, numerical rating scale. Interpretation of Spearman’s correlation coefficient: very weak <0.19; weak 0.2–0.39; moderate 0.4–0.59; strong 0.6–0.79; very strong 0.8–1 [35]. *p* values were <0.0001 in all cases.

**Table 6:** Responsiveness of the UNESP-Botucatu cattle pain scale (UCAPS), cow pain scale (CPS), unidimensional pain scales and rescue analgesia, between the five perioperative time-points, showed as median (minimum–maximum).

	Time-Points					*p*-Value from Fixed Effects		
VARIABLES	M0	M1	M2	M3	M4	Phase	Breed	Evaluator
Rescue Analgesia	0 (0–1) b	0 (0–1) b	1 (0–1) a	1 (0–1) a	1 (0–1) a	0.51	0.62	0.74
NRS	1 (1–6) c	1 (1–6) c	6.5 (1–10) a	6 (1–9) ab	4 (1–10) b	0.29	0.43	**0.03**
VAS	1 (0–57) b	1 (0–55) b	65 (4–99) a	52.5 (0–96) a	38.5 (0–98) a	***p <*** **0.001**	0.70	0.072
**UCAPS items**								
Locomotion	0 (0–2) b	0 (0–2) b	1 (0–2) a	1 (0–2) a	1 (0–2) a	0.90	0.30	**0.02**
Interactive behaviour	0 (0–2) b	0 (0–2) b	1 (0–2) a	1 (0–2) a	1 (0–2) a	0.63	0.56	**0.02**
Activity	0 (0–2) b	0 (0–2) b	2 (0–2) a	2 (0–2) a	2 (0–2) a	0.96	0.65	0.33
Appetite	0 (0–2)	2 (0–2)	2 (0–2)	0 (0–2)	0 (0–2)	0.82	0.80	***p <*** **0.001**
Miscellaneous behaviour	0 (0–2) bc	0 (0–2) c	1 (0–2) ab	1 (0–2) ab	1.5 (0–2) a	0.24	***p <*** **0.001**	0.69
UCAPS total score	2 (0–9) c	3 (0–9) bc	7 (0–10) a	5 (0–10) a	5 (0–10) ab	0.81	0.32	0.97
**CPS items**								
Attention to surroundings	0 (0–1) b	0 (0–1) b	1 (0–1) a	1 (0–1) a	0 (0–1) a	0.63	0.29	0.06
Head position	0 (0–2) bc	0 (0–2) c	1 (0–2) a	1 (0–2) a	0 (0–2) ab	0.89	0.15	***p <*** **0.001**
Ear position	0 (0–2) b	0 (0–2) b	1 (0–2) a	1 (0–2) a	1 (0–2) a	0.35	0.20	0.04
Facial expression	0 (0–1) c	0 (0–1) bc	1 (0–1) a	0.5 (0–1) ab	0 (0–1) ab	0.75	0.23	0.09
Response to approach	1 (0–2)	1 (0–2)	1 (0–2)	1 (0–2)	1 (0–2)	0.74	0.93	0.77
Back position	0 (0–1) b	0 (0–2) ab	0 (0–2) a	0 (0–2) a	0 (0–2) ab	0.09	**0.01**	**0.01**
CPS total score	1 (0–6) d	1 (0–7) cd	5 (0–10) a	3 (0–9) ab	3 (0–7) bc	0.54	0.61	***p <*** **0.001**

Different lowercase letters indicate statistical difference over the time-points (a > b > c > d); multiple comparisons were conducted by linear or general mixed models with post-test corrected by Bonferroni procedure (*p* < 0.05). Bold results represent *p* < 0.05. Rescue analgesia was indicated based in the evaluator’s experience answering a question before assessing the pain scales ‘Do you think it is necessary to provide rescue analgesia?’ yes (1) or no (0). Scales: UCAPS (0-10); CPS (0-10); NRS, Numerical Rating Scale (0-10); VAS, Visual Analogue Scale (0-100). Time-points: M0, 48 hours before surgery and before fasting; M1, before sedation, during fasting (48h after M0); M2, after surgery, three hours after animals were in sternal recumbency, followed by analgesia with morphine; M3, one hour after morphine; M4, 24 h after the end of surgery.

**Table 7:** Internal consistency and item-total correlation of the UNESP-Botucatu cattle pain scale (UCAPS) and cow pain scale (CPS) for all time-points grouped.

Variables	Item-Total (Spearman)	Internal Consistency (Cronbach’s Alpha Coefficient)	Internal Consistency (McDonald’s Omega Coefficient)
**Full-scale UCAPS**	-	**0.72**	**0.79**
**Full-Scale CPS**	-	**0.79**	**0.88**
	**Excluding each item bellow**		
**UCAPS items**			
Locomotion	**0.67**	0.62	**0.72**
Interactive behaviour	**0.69**	0.62	**0.70**
Activity	**0.54**	0.65	**0.76**
Appetite	0.28	**0.76**	**0.83**
Miscellaneous behaviour	**0.39**	**0.71**	**0.78**
**CPS items**			
Attention towards surroundings	**0.68**	**0.74**	**0.84**
Head position	**0.61**	**0.74**	**0.85**
Ear position	**0.63**	**0.74**	**0.85**
Facial expression	**0.54**	**0.76**	**0.86**
Response approach	**0.46**	**0.79**	**0.87**
Back position	**0.51**	**0.79**	**0.88**

Interpretation of Spearman’s rank correlation coefficient (r): 0.3–0.7: acceptable values in bold [16]. Interpretation of the Cronbach’s α coefficient values: 0.60–0.64 minimally acceptable; 0.65–0.69 acceptable; 0.70–0.74 good; 0.75–0.80 very good; >0.80 excellent [39]; bold values >0.70. McDonald’s omega coefficient interpretation: 0.65–0.80, acceptable; >0.80 strong reliability evidence [37]; bold values >65.

**Table 8:** Specificity and sensitivity and its 95% confidence interval of the UNESP-Botucatu cattle pain scale (UCAPS) and cow pain scale (CPS).

Variables	Specificity (%)	Min	Max	Sensitivity (%)	Min	Max
**Full-Scale UCAPS**	**81**	71	89	**87**	76	95
**Full-Scale CPS**	**85**	73	92	**82**	71	89
**UCAPS items**						
Locomotion	**80**	70	88	**81**	69	89
Interactive behaviour	**86**	76	93	**79**	68	87
Activity	**71**	61	80	**78**	64	88
Appetite	63	51	74	48	34	61
Miscellaneous behaviour	60	49	70	**72**	72	81
**CPS items**						
Attention towards surroundings	**85**	74	92	**78**	68	87
Head position	**71**	60	81	**76**	66	84
Ear position	**77**	66	85	**77**	67	86
Facial expression	**88**	78	95	**77**	66	87
Response to approach	64	50	77	**87**	47	99
Back position	**80**	62	92	56	47	66

Interpretation of specificity and sensitivity: excellent 95–100%; good 85–94.9%; moderate 70–84.9%; not specific or sensitive <70%; bold values ≥ 70% [16].

**Table 9:** Rescue analgesia scores, specificity, sensitivity and Youden index of the UNESP-Botucatu cattle pain scale (UCAPS), cow pain scale (CPS) and unidimensional pain scales.

Scale (Min–Max)	Score	Specificity	Sensitivity	Youden Index
UCAPS (0–10)	5	0.84	0.90	0.75
CPS (0–10)	3	0.84	0.90	0.75
NRS (1–10)	4	0.91	1	0.91
VAS (0–100)	32	0.94	0.99	0.94

Scales: NRS, numerical rating scale; VAS, visual analogue scale.

## Data Availability

The data presented in this study are available in the Supplementary Material according to “MDPI Research Data Policies”.

## Supplementary Materials

The following supporting information can be downloaded at: https://www.mdpi.com/article/10.3390/ani13030364/s1, Table S1: Unesp-Botucatu cattle pain scale (UCAPS) [10]; Table S2: Cow pain scale (CPS) [11]; Table S3: Findings from mixed model of the Unesp-Botucatu Cattle Pain Scale for recognizing as the predictor variable; Table S4: Findings from mixed model of the Unesp-Botucatu Cattle Pain Scale for recognizing as the predictor variable.
