# Supplementary material for: Reliability and Validity of UNESP-Botucatu Cattle Pain Scale and Cow Pain Scale in Bos taurus and Bos indicus Bulls to Assess Postoperative Pain of Surgical Orchiectomy
Source: Animals (Basel). 2023 Jan 20;13(3):364. doi: 10.3390/ani13030364 (PMC9913732; doi:10.3390/ani13030364)
Supplement: Supplementary file 1 [file animals-13-00364-s001.zip › supplementary.material.animals_corrected_RM.pdf]

animals MDPI

Table S1. Unesp-Botucatu Cattle Pain Scale (UCAPS) [10]

Item	Description
Locomotion	(0) Walking with no obviously abnormal gait.<br>(1) Walking with restriction, may be with hunched back and/or short steps.<br>(2) Reluctant to stand up, standing up with difficulty or not walking.
Interactive behaviour	(0) Active; attention to tactile and/or visual and/or audible environmental stimuli; when near other animals, can interact with and/or accompany the group.<br>(1) Apathetic: may remain close to other animals but interacts little when stimulated.<br>(2) Apathetic: may be isolated or may not accompany the other animals; does not react to tactile, visual and/or audible environmental stimuli.
Activity	(0) Moves normally.<br>(1) Restless, moves more than normal or lies down and stands up with frequency.<br>(2) Moves less frequently in the pasture or only when stimulated.
Appetite	(0) Normorexia and/or rumination.<br>(1) Hyporexia.<br>(2) Anorexia.
Miscellaneous behaviours	Wagging the tail abruptly and repeatedly.<br>Licking the affected area.<br>Moves and arches the back when in standing posture.<br>Kicking/foot stamping.<br>Hind limbs extended caudally when in standing posture.<br>Head below the line of spinal column.<br>Lying down in ventral recumbency with full or partial extension of one or both hind limbs.<br>Lying down with the head on/close to the ground.<br>Extends the neck and body forward when lying in ventral recumbency.<br>(0) All of the above-described behaviours are absent.<br>(1) Presence of 1 of the behaviours described above.<br>(2) Presence of 2 or more of the behaviours described above.

Table S2. Cow pain scale (CPS) [11]

Item	Description
Attention towards the surroundings	(0) Active and attentive. The cow is active: eating, ruminating, grooming etc. The cow is attentive and/or attention seeking/curious.<br>(1) Quiet/depressed. The cow is not active, avoiding eye contact, may move away from the observer.
Head position	(0) High/level of withers. The cow is active, eating, ruminating or is contact seeking/curious.<br>(1) Level of withers. The cow is not active, not eating, ruminating, grooming, or sleeping.<br>(2) Low. The cow is not active, not eating, ruminating, grooming or sleeping; may lie down quickly after getting up.
Ear position	(0) Both ears forward or one ear forward or back and the other listening.<br>(1) Ears back/asymmetric ear movements. Both ears back or moving in different directions (not forward or back).<br>(2) Lambs' ears. Both ears to the sides and lower than usual; the pinna facing slightly down.
Facial expression	(0) Attentive/neutral look. The cow is attentive, focused on a task (eating, ruminating) or sleeping<br>(1) Tense expression/strained appearance. The cow has a worried or strained look, furrows above the eyes and puckers above the nostrils.
Response to approach	(0) Look at observer, head up, ears forward or occupied with activity (grooming, ruminating).<br>(1) Look at observer, ears not forward, leave when approached.<br>(2) May/may not look at observer, head low, ears not forward may leave slowly.
Back position	(0) Normal.<br>(1) Slightly arched back.<br>(2) Arched back.

Table S3. Findings from mixed model of the Unesp-Botucatu Cattle Pain Scale for recognizing as the predictor variable.

Parameters	Angular coefficient (beta)			
**Fixed effects**	**Estimated**	**SE**	**T-value**	***p*-value**
Intercept (alpha)	2.6342	0.5725	4.6010	1.27-5
Evaluator	0.0053	0.1859	0.0280	0.9775
Time-point M1	0.7237	0.7157	1.0110	0.3147
Time-point M2	3.7105	0.7157	5.1840	1.35-6
Time-point M3	2.7632	0.7157	3.8610	2.14-4
Time-point M4	2.4605	0.7157	3.4380	8.94-4
Phase2	-0.0368	0.1559	-0.2360	0.8134
Breed Nelore	-0.4500	0.4533	-0.9930	0.3235

Random effects

Parameters		Variance	SD	Level number	ICC
Evaluator:(Time-point: Animal)	(Intercept)	0.4879	0.6985	190	0.07
Time-point: Animal	(Intercept)	4.046	2.011	95	0.59
Animal	(Intercept)	2.420-10	1.556-5	19	0.00
Residual		2.309	1.519		

Model parameters	Mixed model	Null mixed model	Linear model	Null linear model	*p*-value
Degree of freedom	12	5	9	2	
Log-Likelihood	-812.57	-829.20	-895.4262	-944.3764	2.2-16
AIC	1649.150	1668.396	1808.852	1892.753	
BIC	1696.432	1688.097	1844.314	1900.633	
F-statistic (p-value)	NA	NA	15.62 (2.2-16)	0.00 (0.00)	
Adjusted R2 (R2)	NA	NA	0.21 (0.23)	0.00 (0.00)	
Pseudo R2 (total)	0.74	0.73	NA	NA	
Pseudo R2 (fixed effects)	0.22	0.00	NA	NA	

Legend: SE, Standard error; SD, Standard deviation; ICC, Intra-class correlation coefficient; NA, Not applicable.

Table S4. Findings from mixed model of the Unesp-Botucatu Cattle Pain Scale for recognizing as the predictor variable.

**Parameters**	**Angular coefficient (beta)**			
**Fixed effects**	**Estimated**	**SE**	**T-value**	***p*-value**
Intercept (alpha)	1.0386	0.4469	2.3240	0.0227
Evaluator	0.7368	0.1813	4.0650	9.97-5
Time-point M1	0.1842	0.5303	0.3470	0.7293
Time-point M2	3.4868	0.5303	6.5750	6.64-9
Time-point M3	2.1842	0.5303	4.1190	0.0001
Time-point M4	1.4868	0.5303	2.8040	0.0065
Phase 2	-0.0842	0.1391	-0.6050	0.5456
Breed Nelore	-0.1933	0.3753	-0.5150	0.6131

Random effects

**Parameters**		**Variance**	**SD**	**Level number**	**ICC**
Evaluator:(Time-point: Animal)	(Intercept)	0.6419	0.8012	190	0.14
Time-point: Animal	(Intercept)	1.8911	1.3752	95	0.42
Animal	(Intercept)	0.1330	0.3647	19	0.03
Residual		1.8377	1.3556		

**Model parameters**	**Mixed model**	**Null mixed model**	**Linear model**	**Null linear model**	***p*-value**
Degree of freedom	12	5	9	2	
Log-Likelihood	-763.81	-793.81	-816.68	-883.80	2.78-9
AIC	1551.628	1597.622	1651.369	1771.606	
BIC	1598.910	1617.323	1686.831	1779.487	
F-statistic (p-value)	NA	NA	22.52 (2.2-16)	0.00 (0.00)	
Adjusted R2 (R2)	NA	NA	0.28 (0.30)	0.00 (0.00)	
Pseudo R2 (total)	0.71	0.70	NA	NA	
Pseudo R2 (fixed effects)	0.29	0.00	NA	NA	

Legend: SE, Standard error; SD, Standard deviation; ICC, Intra-class correlation coefficient; NA, Not applicable
